# Sustainable Hand Surgery: Incorporating Water Efficiency Into Clinical Practice

**DOI:** 10.7759/cureus.38331

**Published:** 2023-04-30

**Authors:** Sophie Gasson, Francesca Solari, Edwin P Jesudason

**Affiliations:** 1 Medicine, Cardiff University, Cardiff, GBR; 2 Trauma and Orthopaedics, Ysbyty Gwynedd, Bangor, GBR

**Keywords:** orthopaedics, sustainability, water reduction, water waste, water use

## Abstract

Introduction

Public health and well-being outcomes are intimately connected with the health of our planet. Climate change has numerous far-reaching effects. Managing and mitigating these risks to human health presents one of the next challenges to global healthcare. The current usage of planetary resources is unsustainable. Surgical procedures are particularly resource-intensive, often utilising vast amounts of single-use consumables, like water. In the last 100 years global usage of fresh water has increased six-fold and continues to rise by 1% year on year. It is well established that initial hand sterilization and maintenance of hand sterility during the surgical list are essential for preventing hospital-acquired infections and associated morbidity and mortality. This study aims to estimate the current daily water usage of two typical hand surgery lists from a District General Hospital in North Wales, to determine potential water savings by switching exclusively to an alcohol-based hand rub for subsequent scrubs, in line with current national guidelines.

Methods

Observational study estimation of water consumption from a temperature-controlled manual tap required using a 1 litre volumetric jug where the time taken to fill was recorded. Three separate observational samples were taken, and a mean was calculated. This mean determined the amount of water dispensed from the tap in a standard 3 min scrub and subsequent 1 min scrub. Two different theatre schedules were analysed: 1. A trauma list (five cases) and 2. A higher volume minor elective procedure schedule (16 cases), in this case a wide-awake local anaesthetic no tourniquet (WALANT) carpal tunnel release (CTR).

Results

Each case regardless of procedure had approximately three persons scrubbed. 20.57L of water is used for one person to scrub for 3 mins and an extra 6.8574L for each subsequent 1 min scrub. Therefore, current daily water consumption could reach 143.99L during the major hand trauma list and 411.4L during a high-volume carpal tunnel release list.

Conclusion

Simply following current guidelines by switching to alcohol-based hand rub just for subsequent scrubs could reduce water consumption by 57.2% for hand trauma lists and 70.2% for high-volume CTR lists.

## Introduction

Public health and well-being outcomes are intimately connected with the health of our planet with numerous studies identifying the harmful and far-reaching effects of climate change [1,2]. Managing and mitigating these risks to human health presents a significant challenge to global healthcare [3]. It is well established that initial hand sterilization and maintenance of hand sterility during the surgical list are essential for preventing hospital-acquired infections and associated morbidity and mortality [4,5]. In 2019, the Welsh Government declared a climate emergency, triggering the National Health Service (NHS) in Wales to pledge to reduce total emissions by one-third by 2030 [6]. In 2018, NHS Wales generated a total carbon footprint of 1,001,378 tCO2e with waste production accounting for 695 tCO2e [7]. The current usage of planetary resources is unsustainable. In the last 100 years global usage of fresh water has increased six-fold and continues to rise by 1% year on year [8]. Surgical procedures are particularly resource-intensive, often utilising vast amounts of single-use consumables, like water. Not only are the environmental issues of concern here there are financial implications to consider also. The NHS is a publicly funded entity with finite financial resources. NHS Wales was in over £47 million of debt in 2020-21 [9]. Put into perspective; financial savings need to be made alongside improvements in environmental sustainability to promote overall sustainability in the service provided.

Hand sterilization and maintenance of hand sterility during the surgical list are essential for preventing hospital-acquired infections and associated morbidity and mortality. The National Institute for Clinical Excellence (NICE) guidelines for hand decontamination (2014) recommends two scenarios whereby soap and water must be used over alcohol hand rub; when there is visible soiling or potential contact with bodily fluids and when caring for patients with vomiting or diarrhoeal illness. Operating theatre-specific guidance states that staff should wash hands with an aqueous antiseptic surgical solution prior to the first operation and may subsequently use alcoholic hand rub; unless hands are visibly soiled [5]. A typical standard surgical scrub regimen is an initial 3 min scrub with an aqueous antiseptic solution followed by a 1 min scrub thereafter unless obviously soiled [5]. Hand washing with soap is known to be more effective than with water alone; the presence of bacteria on hands is significantly reduced with soap and water compared to water itself in one trial [10] and in another reducing rates of pneumonia, diarrhoeal illness and impetigo [11].

Carpal tunnel syndrome whereby the median nerve is compressed within the finite carpal tunnel is the commonest peripheral nerve entrapment in the UK with a prevalence of up to 16% [12]. Subsequently, carpal tunnel release (CTR) is a common procedure [13]. CTR is also quick to complete in around 30 mins of theatre time [14]. Quick turn-around high-volume surgical lists require multiple scrubs that consume lots of water. This project aims to assess how much water is consumed in a high-volume CTR list compared to a hand trauma list typically with fewer cases.

The aims of the current study are assessment of water consumption in two different hand surgery lists and estimation of the potential financial savings with the use of an alcohol-based hand gel for surgical hand preparation.

## Materials and methods

Estimating water consumption from a temperature-controlled manual tap in the theatre was performed with a 1-litre volumetric jug and recording the time taken to fill. Three separate observational samples were taken, and a mean was calculated. The mean was then extrapolated to determine the amount of water dispensed from the tap in a standard 3 min scrub and subsequent 1 min scrub. Two different theatre schedules were analysed; a hand trauma list and a higher volume minor elective procedure schedule, in this case, a wide-awake local anaesthetic no tourniquet (WALANT) carpal tunnel release (CTR) list. Data regarding the numbers of operations performed in Ysbyty Gwynedd was then retrospectively collected for the year 2022. This allowed estimations of water consumption across the department for a whole year to be estimated.

This was a quality improvement study. Ethical approval was not required as no patient information was collected.

## Results

A full day of hand trauma procedures had on average five cases and the higher volume list for CTR only had on average 16 cases in our hospital in 2022. Each case regardless of procedure had three to four persons scrubbing, these included a lead surgeon, surgical assistant, scrub nurse and/or a medical or nursing student.

The mean time to fill a 1-litre jug is 8.75s, making the average tap flow rate 0.11429L/s. Therefore, it will take 20.57L of water for one person to scrub for 3 mins and an extra 6.8574L for each subsequent 1 min scrub.

Water consumption - hand trauma list (five cases)

Three people scrubbing initially for 3 mins equates to 61.71L. If subsequent scrubs were 1 min each this adds a further 20.57L per case, making the total litres of water consumed for one day 143.99L (for three people scrubbed for each of the five cases). Each person scrubbed using 47.96L per day.

Water consumption - CTR list (16 cases)

The 16 cases are split into two sessions, eight cases in each. An initial 3 min scrub before the first case in each session is standard. Ideally, then there are two 3 mins scrubs and 14 1 min scrubs throughout the day. Each session, therefore, consumes 205.7L for three people making a total of 411.4L for the day. The elective high-volume case list had fewer delays making this list much more scrubbing efficient.

Potential water conservation

Switching to an alcohol-based hand rub for subsequent scrubs instead of using water would save 82.29L per day for a major trauma list and 288.7L per day for a CTR list when taking into account three people scrubbing per case. It is also important to note that often only the surgeon and assistant will perform the shorter scrub as they are involved in each case, the scrub nurse team rotating who scrubs for each case. Table 1 below summarises all of the estimated water usage and potential savings.

**Table 1:** Calculated water use for two different hand surgery lists and potential water savings

	Hand Trauma List	Carpal Tunnel Release List
Number of Cases (Average)	5	16
Litres of water used per person per day	48.00	137.04
Litres of water used for three people scrubbed per day	143.99	411.4
Potential Litres of water saved per person per day	27.43	96.26
Potential litres of water saved for three people scrubbed per day	82.29 (57.2% saving)	288.77 (70.2% saving)

A retrospective analysis of operating lists in 2022 showed 2178 operations in total. On the daily trauma list, there were 1071 cases, in one elective theatre there were 495 cases and in our second elective theatre there were 612. Taking this into consideration, with our estimated calculations Ysbyty Gwynedd used 134,404.38 litres of water in scrubbing for trauma and orthopaedic lists in 2022. This number was calculated by counting the number of operations in total in the year and estimating three people scrubbed for each case. Of course, this is an estimation, as a number of cases may not require staff to scrub; for example manipulations under anaesthetic.

The cost of surgical hand preparation is also a factor to consider here. The two options available in Ysbyty Gwynedd are 4% chlorhexidine and 7.5% iodine scrubs. NHS procurement costs these at £5.55 and £9.97 respectively. Alcohol-based hand gel for surgical preparation is more expensive per 500ml bottle at £16.92. Based on volumes per scrub chlorhexidine is the cheapest and iodine is the most expensive. This is summarised in Table 2.

**Table 2:** Cost analysis of surgical hand preparation

Hand Preparation	Cost 500ml	Cost per scrub (15 ml and 6ml)	Scrubs per 500ml bottle	Cost per hand trauma list per person	Cost per carpal tunnel decompression list per person
Chlorhexidine	£5.55	17p	33	68p	£2.38
Iodine	£9.97	30p	33	£1.20	£4.20
Alcohol-based gel	£16.92	20p	83	80p	£2.80

## Discussion

These results show that the hand theatre list consumes significant amounts of water. Overall in 2022 in Ysbyty Gwynedd 134,404.38 litres of water were estimated to be used in surgical hand preparation. We have shown that there are significant variations in water use depending on the make-up of the list; a WALANT high-volume carpal tunnel decompression list utilising significantly more water than a more standard hand trauma list given the number of cases per list. Our findings of a 3-min scrub requiring 20.57 litres of water are similar to other studies looking at water sustainability, where estimates have ranged from 15.9L in a 2-min scrub [15] to 18.5L [16] and 20.2L [17]. We can be reassured that our calculations are reliable estimates of consumption.

Making a simple switch to an alcohol-based hand preparation gel could reduce water consumption by 57% for hand trauma lists and up to 70% for high-volume CTR lists. Following the current guidelines [4] for hand sterilisation could have profound differences in the consumption of single-use utilities in hand surgery. Updating hand sterilization techniques is not unique to hand surgery, it is applicable to all surgical specialties, which could improve the overall water consumption of the hospital.

The benefits of using alcohol-based hand rub include a quicker and more efficient “time to table” preparation and prevention of epithelial lipid barrier deterioration from over-washing [15]. Evidence also demonstrates the equivalence in sterility quality and duration for alcohol-based hand rub in comparison to soap and water [17-23]. Evidence surrounding the effectiveness of surgical alcoholic hand rubs versus traditional scrubs is variable in quality as noted in the most recent Cochrane Review on the area [24]. The studies are highly variable in their methods, however, this review did find that there was no clear evidence of the inferiority of an alcoholic hand rub compared to traditional scrubs. It also found there was possibly better performance in terms of the formation of colony-forming units of bacteria for the alcohol-based rubs, though the clinical significance for this in terms of surgical site infection was debatable. Level 1 evidence here demonstrating clear benefit or non-inferiority of alcohol-based rubs for surgical hand preparation is needed to further the claim that it is safe to make this change. Studies have also demonstrated surgeon preference for alcohol-based gel; in terms of colour, smell, texture, skin dryness, and ease of application [25,26]. Both NICE and the WHO have stated that alcohol-based hand rubs are suitable for surgical hand preparation [4,5] but the WHO has similarly commented on the need for greater quality clinical trials in the area and for this to be in much larger volumes also [5].

We have calculated an estimated water volume saving of between 57.2 and 70.2% for our hand surgery lists in Ysbyty Gwynedd. When looking at the costs of surgical hand preparation, for a hand trauma list the cost of chlorhexidine for three people scrubbing would be £2.04, iodine £3.60, and for alcohol rub £2.40. For the higher volume list, this would be £7.14, £12.60, and £8.40 for three people to scrub with chlorhexidine, iodine, and alcohol, respectively. The alcohol gel is more expensive than chlorhexidine but cheaper than iodine per scrub. Until assessments are made of surgeon and scrub staff preference for chlorhexidine versus iodine in our department it is uncertain what the cost savings could be. But alongside the significant savings in water, the use of alcohol hand preparation appears not to be a significant financial hurdle to overcome.

NHS England spent £89.1mn on water in 2022, using 35.2 million m3 [27] and NHS Wales spent £37.1mn on water in 2018 [28]. Water is a significant cost in NHS Wales and with the deficit in the budget in 2021-22 being £47mn the importance of financial sustainability becomes clearer. Whilst we cannot accurately assess the costs here, only estimations of water use, showing that we could save 57.2% to 70.2% of water in surgical scrubbing for hand surgery lists is potentially significant in helping to promote not only environmental but also financial sustainability in NHS Wales. Water use in Ysbyty Gwynedd in 2021-22 was metered at 159,613m3. Whilst it isn't possible to subdivide this water use across departments in the hospital, this gives an estimate of how much could be saved. In Ysbyty Gwynedd two orthopaedic lists run per day from Monday to Friday and one trauma list per day at the weekend. Conservative estimates of 82.29L saved per day for 250 working days a year and 104 weekend days comes to 59,931.16L per year saved, or 59.93m3.

Barriers to the implementation of an alcohol-based gel and local policy change include time constraints [29], particularly in an already stretched service, staff variations in scrubbing preferences, and individual opinions of best practice. Implementing changes across the different orthopaedic subspecialties could be challenging, especially within the arthroplasty subspecialty, given the significant morbidity associated with prosthetic joint infection. Level 1 evidence would need to be published to safely help arthroplasty surgeons make the swap to an alcohol-based gel, to be confident the risk of infection is not inferior with this product. Currently, there is one randomised study in elective orthopaedics [23] which has shown no difference in the number of colony-forming units in alcohol-based rubs compared to standard scrubs, but this was not corroborated with an assessment of infection rates in the patients post-operatively. More research will need to be done to show at least non- inferiority of alcohol-based hand preparation compared to standard hand preparation.

Thinking forwards and where we go with this research, we have demonstrated that significant savings in the use of water could be achieved with a simple switch to an alcohol-based rub for subsequent hand preparations after an initial scrub. However, the next steps here should be to ascertain the risk profile from this switch using a prospective study in our hand trauma and CTR lists, assessing the costs then of using such hand preparation techniques and assessing infection rates, costs of treatment and follow-ups. Not only does the financial implication matter in the purchasing and procurement of the hand preparation, but also in the prescription of antibiotics and clinic visits for follow-up. If we want to improve the sustainability of our hand trauma and CTR lists we will need to consider the change beyond the operation itself and think about the post-operative implications not assessed in the current study.

The biggest limitation of this study is the small sample and limited study design, using a consultant surgeon’s typical operating lists within a single theatre in a single district general hospital. In addition, the carbon footprint generated by heating the water onsite and upstream carbon emissions from the collection, sterilization, transportation, and recycling processes of the water provider was beyond the scope of this project. However, Dwr Cymru provides water utility to Wales, as a result, is one of the largest energy users in the country. The annual performance report 2020/21, states 23% of their total energy usage comes from green renewable sources, falling short of the 31% target for self-sufficiency [30]. By addressing water efficiency in the setting of hand surgery we could contribute to improvements in water sustainability and promote making sustainable improvements in orthopaedic surgery and beyond.

## Conclusions

This study has demonstrated an estimation of water use in hand trauma and carpal tunnel release lists. This simple analysis has highlighted the potential water and cost savings that could be achieved by switching to an alcohol-based gel hand preparation. Alongside other possible interventions, like, automated foot pedals and taps, significant savings in water use could be achieved.
